# Supplementary material for: Ensembler: Enabling High-Throughput Molecular Simulations at the Superfamily Scale
Source: PLoS Comput Biol. 2016 Jun 23;12(6):e1004728. doi: 10.1371/journal.pcbi.1004728 (PMC4918922; doi:10.1371/journal.pcbi.1004728)
Supplement: S1 Text — (DOCX) [file pcbi.1004728.s001.docx]

*Human Abl1 sequence*

1 MLEICLKLVG CKSKKGLSSS SSCYLEEALQ RPVASDFEPQ GLSEAARWNS KENLLAGPSE 60

61 NDPNLFVALY DFVASGDNTL SITKGEKLRV LGYNHNGEWC EAQTKNGQGW VPSNYITPVN 120

121 SLEKHSWYHG PVSRNAAEYL LSSGINGSFL VRESESSPGQ RSISLRYEGR VYHYRINTAS 180

181 DGKLYVSSES RFNTLAELVH HHSTVADGLI TTLHYPAPKR NKPTVYGVSP NYDKWEMERT 240

241 DITMKHKLGG GQYGEVYEGV WKKYSLTVAV KTLKEDTMEV EEFLKEAAVM KEIKHPNLVQ 300

301 LLGVCTREPP FYIITEFMTY GNLLDYLREC NRQEVNAVVL LYMATQISSA MEYLEKKNFI 360

361 HRDLAARNCL VGENHLVKVA DFGLSRLMTG DTYTAHAGAK FPIKWTAPES LAYNKFSIKS 420

421 DVWAFGVLLW EIATYGMSPY PGIDLSQVYE LLEKDYRMER PEGCPEKVYE LMRACWQWNP 480

481 SDRPSFAEIH QAFETMFQES SISDEVEKEL GKQGVRGAVS TLLQAPELPT KTRTSRRAAE 540

541 HRDTTDVPEM PHSKGQGESD PLDHEPAVSP LLPRKERGPP EGGLNEDERL LPKDKKTNLF 600

601 SALIKKKKKT APTPPKRSSS FREMDGQPER RGAGEEEGRD ISNGALAFTP LDTADPAKSP 660

661 KPSNGAGVPN GALRESGGSG FRSPHLWKKS STLTSSRLAT GEEEGGGSSS KRFLRSCSAS 720

721 CVPHGAKDTE WRSVTLPRDL QSTGRQFDSS TFGGHKSEKP ALPRKRAGEN RSDQVTRGTV 780

781 TPPPRLVKKN EEAADEVFKD IMESSPGSSP PNLTPKPLRR QVTVAPASGL PHKEEAGKGS 840

841 ALGTPAAAEP VTPTSKAGSG APGGTSKGPA EESRVRRHKH SSESPGRDKG KLSRLKPAPP 900

901 PPPAASAGKA GGKPSQSPSQ EAAGEAVLGA KTKATSLVDA VNSDAAKPSQ PGEGLKKPVL 960

961 PATPKPQSAK PSGTPISPAP VPSTLPSASS ALAGDQPSST AFIPLISTRV SLRKTRQPPE 1020

1021 RIASGAITKG VVLDSTEALC LAISRNSEQM ASHSAVLEAG KNLYTFCVSY VDSIQQMRNK 1080

1081 FAFREAINKL ENNLRELQIC PATAGSGPAA TQDFSKLLSS VKEISDIVQR 1130

*Sequences for human and chicken Src, aligned using Clustal Omega*

SRC_HUMAN 1 MGSNKSKPKD ASQRRRSLEP AENVHGAGGG AFPASQTPSK PASADGHRGP SAAFAPAAAE 60

SRC_CHICK 1 MGSSKSKPKD PSQRRRSLEP PDSTH---HG GFPASQTPNK TAAPDTHRTP SRSFGTVATE 57

***.****** ********* :..* * .*******.* *: * ** * * :*. .*:*

SRC_HUMAN 61 PKLFGGFNSS DTVTSPQRAG PLAGGVTTFV ALYDYESRTE TDLSFKKGER LQIVNNTEGD 120

SRC_CHICK 58 PKLFGGFNTS DTVTSPQRAG ALAGGVTTFV ALYDYESRTE TDLSFKKGER LQIVNNTEGD 117

********:* ********** ********* ********** ********** **********

SRC_HUMAN 121 WWLAHSLSTG QTGYIPSNYV APSDSIQAEE WYFGKITRRE SERLLLNAEN PRGTFLVRES 180

SRC_CHICK 118 WWLAHSLTTG QTGYIPSNYV APSDSIQAEE WYFGKITRRE SERLLLNPEN PRGTFLVRES 177

*******:** ********** ********** ********** ******* ** **********

SRC_HUMAN 181 ETTKGAYCLS VSDFDNAKGL NVKHYKIRKL DSGGFYITSR TQFNSLQQLV AYYSKHADGL 240

SRC_CHICK 178 ETTKGAYCLS VSDFDNAKGL NVKHYKIRKL DSGGFYITSR TQFSSLQQLV AYYSKHADGL 237

********** ********** ********** ********** ***.****** **********

SRC_HUMAN 241 CHRLTTVCPT SKPQTQGLAK DAWEIPRESL RLEVKLGQGC FGEVWMGTWN GTTRVAIKTL 300

SRC_CHICK 238 CHRLTNVCPT SKPQTQGLAK DAWEIPRESL RLEVKLGQGC FGEVWMGTWN GTTRVAIKTL 297

*****.**** ********** ********** ********** ********** **********

SRC_HUMAN 301 KPGTMSPEAF LQEAQVMKKL RHEKLVQLYA VVSEEPIYIV TEYMSKGSLL DFLKGETGKY 360

SRC_CHICK 298 KPGTMSPEAF LQEAQVMKKL RHEKLVQLYA VVSEEPIYIV TEYMSKGSLL DFLKGEMGKY 357

********** ********** ********** ********** ********** ****** ***

SRC_HUMAN 361 LRLPQLVDMA AQIASGMAYV ERMNYVHRDL RAANILVGEN LVCKVADFGL ARLIEDNEYT 420

SRC_CHICK 358 LRLPQLVDMA AQIASGMAYV ERMNYVHRDL RAANILVGEN LVCKVADFGL ARLIEDNEYT 417

********** ********** ********** ********** ********** **********

SRC_HUMAN 421 ARQGAKFPIK WTAPEAALYG RFTIKSDVWS FGILLTELTT KGRVPYPGMV NREVLDQVER 480

SRC_CHICK 418 ARQGAKFPIK WTAPEAALYG RFTIKSDVWS FGILLTELTT KGRVPYPGMV NREVLDQVER 477

********** ********** ********** ********** ********** **********

SRC_HUMAN 481 GYRMPCPPEC PESLHDLMCQ CWRKEPEERP TFEYLQAFLE DYFTSTEPQY QPGENL 536

SRC_CHICK 478 GYRMPCPPEC PESLHDLMCQ CWRKDPEERP TFEYLQAFLE DYFTSTEPQY QPGENL 533

********** ********** ****:***** ********** ********** ******

**S1 Text**. **Sequences and residue numbering schemes for Src and Abl1.** Kinase catalytic domains are highlighted in red, and the conserved residues analyzed in the main text (Figs. 8 and 9) are highlighted with yellow background.
